# Permeability–diffusivity modeling vs. fractional anisotropy on white matter integrity assessment and application in schizophrenia^☆^

**DOI:** 10.1016/j.nicl.2013.06.019

**Published:** 2013-07-11

**Authors:** P. Kochunov, J. Chiappelli, L.E. Hong

**Affiliations:** a Maryland Psychiatric Research Center, Department of Psychiatry, University of Maryland School of Medicine, Baltimore, USA; b Department of Physics, University of Maryland Baltimore County, USA

**Keywords:** DTI, Axonal integrity, Q-space, Permuability-diffusivity index, Fractional anisotropy, Schizophrenia

## Abstract

**Introduction:**

Diffusion tensor imaging (DTI) assumes a single pool of anisotropically diffusing water to calculate fractional anisotropy (FA) and is commonly used to ascertain white matter (WM) deficits in schizophrenia. At higher b-values, diffusion-signal decay becomes bi-exponential, suggesting the presence of two, unrestricted and restricted, water pools. Theoretical work suggests that semi-permeable cellular membrane rather than the presence of two physical compartments is the cause. The permeability–diffusivity (PD) parameters measured from bi-exponential modeling may offer advantages, over traditional DTI-FA, in identifying WM deficits in schizophrenia.

**Methods:**

Imaging was performed in N = 26/26 patients/controls (age = 20–61 years, average age = 40.5 ± 12.6). Imaging consisted of fifteen b-shells: b = 250–3800 s/mm^2^ with 30 directions/shell, covering seven slices of mid-sagittal corpus callosum (CC) at 1.7 × 1.7 × 4.6 mm. 64-direction DTI was also collected. Permeability–diffusivity-index (PDI), the ratio of restricted to unrestricted apparent diffusion coefficients, and the fraction of unrestricted compartment (M_u_) were calculated for CC and cingulate gray matter (GM). FA values for CC were calculated using tract-based-spatial-statistics.

**Results:**

Patients had significantly reduced PDI in CC (p ≅ 10^− 4^) and cingulate GM (p = 0.002), while differences in CC FA were modest (p ≅ .03). There was no group-related difference in M_u_. Additional theoretical-modeling analysis suggested that reduced PDI in patients may be caused by reduced cross-membrane water molecule exchanges.

**Conclusion:**

PDI measurements for cerebral WM and GM yielded more robust patient–control differences than DTI-FA. Theoretical work offers an explanation that patient–control PDI differences should implicate abnormal active membrane permeability. This would implicate abnormal activities in ion-channels that use water as substrate for ion exchange, in cerebral tissues of schizophrenia patients.

## Highlights

• We evaluated diffusion-imaging measurements of membrane permeability.
• Schizophrenia patients had restricted permeability of axonal membranes.
• Significance of permeability measurements were more robust than DTI-FA.
• Restricted permeability in patients may reflect activity molecular ion pumps.

## 1 Introduction

Diffusion tensor imaging (DTI) is widely used for quantification of cerebral white matter (WM) integrity in disorders that affect cerebral connectivity including schizophrenia (Friedman et al., 2008; Jones et al., 2006; Kanaan et al., 2005; Kochunov et al., 2013; Mori et al., 2007; Nazeri et al., 2013) and other psychiatric and neurological disorders (Allan et al., 2011; Blood et al., 2011; Kanaan et al., 2005; Kieseppa et al., 2011; Korgaonkar et al., 2011; White et al., 2008; Zhang et al., 2012). DTI-derived fractional anisotropy (FA) of water diffusion (Basser and Pierpaoli, 1996; Kochunov et al., 2007; Pfefferbaum et al., 2000; Song et al., 2003, 2005) has emerged as one of the more sensitive imaging biomarkers in schizophrenia research (Friedman et al., 2008; Glahn et al., 2011; Mori et al., 2007). Reduced FA values are consistently reported in this disorder (Friedman et al., 2008; Perez-Iglesias et al., 2011) and patients show an accelerated decline with age in FA values, particularly in the genu of the corpus callosum (Kochunov et al., 2013). The biological basis of reduced FA is uncertain, but impaired axonal myelination and/or glial cell deficits are the likely causes (Palaniyappan et al., 2013).

DTI uses a single diffusion weighting b-value and the 3D, multivariate Gaussian model to quantify diffusion behavior of water (Basser and Pierpaoli, 1996). It assumes a single pool of anisotropically diffusing water and uses a mono-exponential function to describe the signal decay due to diffusion weighting. This approximation is successful at modest diffusion weighting (b-values up to ~ 1000 s/mm^2^). At higher diffusion weighting, the diffusion signal becomes a bi-exponential function of b-values, representing two, unrestricted and restricted, diffusion ‘pools’ (Assaf and Cohen, 1998; Clark et al., 2002; Wu et al., 2011a,b). The two exponential components are often ascribed to two physical compartments, typically to extra-and-intracellular compartments (Clark et al., 2002; Wu et al., 2011a,b). However, experiments in animals, extruded liposomes and computer simulations show that population fractions for the unrestricted and restricted ‘pools’ do not coincide with the known volume fractions of extra- and intracellular spaces (Hwang et al., 2003; Schwarcz et al., 2004; Stokes et al., 2012; Yablonskiy et al., 2003). More likely, the bi-exponential function provides a better description of the complex signal decay behavior than the mono-exponential function. Theoretical work by Sukstanskii and colleagues resulted in a model that explained the bi-exponential behavior of diffusion signal by the presence of a permeable cellular membrane, which creates an inhomogeneous distribution of local transverse magnetization (Sukstanskii et al., 2003, 2004). Moreover, the population fractions and diffusion coefficients derived from the bi-exponential modeling were shown to be sensitive to membrane's permeability. Here, we used the theoretical work by Sukstanskii and colleagues for analysis of data collected in human subjects and performed a comparison between FA values and a permeability–diffusivity index (PDI) using schizophrenia as a disease model.

Our experiment was performed in the mid-sagittal band of corpus callosum using a clinical, 3 T scanner. This region was chosen because schizophrenia related differences are consistently observed there, especially in the genu (Henze et al., 2012; Kochunov et al., 2013; Kubicki et al., 2008; Lee et al., 2013). The permeability–diffusivity (PD)-model has not been adequately evaluated on brain areas containing intra-voxel crossing fibers. Corpus callosum has a simple parallel fiber orientation that contains no crossing fibers and is consistent across subjects (Aboitiz et al., 1992). Notably, the PD-modeling can also be applied to gray matter (GM), where diffusion decay is also bi-exponential (Clark et al., 2002). We explored this by fitting PD-model to data collected from the cingulate cortex overlaying the corpus callosum, and examined the group-differences in GM-based PDI between schizophrenia and healthy control. This may provide a diffusion imaging method that can be applied across cerebral tissue boundaries.

## 2 Methods

### 2.1 Subjects

A total of 52 (33 M, age = 20–61 years, average age = 40.5 ± 12.) individuals participated in the study. Half of them (18 males, age = 39.8 ± 12.8 years) were patients diagnosed with schizophrenia and the others (15 males, age 41.2 ± 12.4 years) were healthy controls. Additional clinical and demographic information is provided in Table 1. Patients were recruited through the Maryland Psychiatric Research Center outpatient clinics. Controls were recruited through media advertisements. All subjects were evaluated with Structured Clinical Interview for DSM-IV (First et al. 1997). Patients were those with current Axis I schizophrenia diagnosis. Controls were subjects without Axis I psychiatric diagnosis. Controls could not have current or past Axis I diagnoses. Illicit substance and alcohol abuse and dependence were exclusion criteria. Except three medication-free participants, all schizophrenia patients were on antipsychotic medications. Clinical symptoms in patients were measured by the 20-item Brief Psychiatric Rating Scale (BPRS), using a score of 1–7 on each item. There were no significant differences in the age and body-mass index (BMI) between patients and controls. The exclusion criteria included any major neurological diagnosis or events such as head trauma, seizure, stroke or transient ischemic attack.

## 3 Imaging and data analysis protocols

All imaging was performed at the University of Maryland Center for Brain Imaging Research using a Siemens 3 T TRIO MRI (Erlangen, Germany) system and 32 channel phase array head coil. Imaging for each subject was performed on two separate days. The multi-b-value diffusion imaging (MBI) data were collected in one session while the high-angular resolution diffusion imaging (HARDI) data were collected during the other.

### 3.1 MBI protocol

**Fig. 1:**
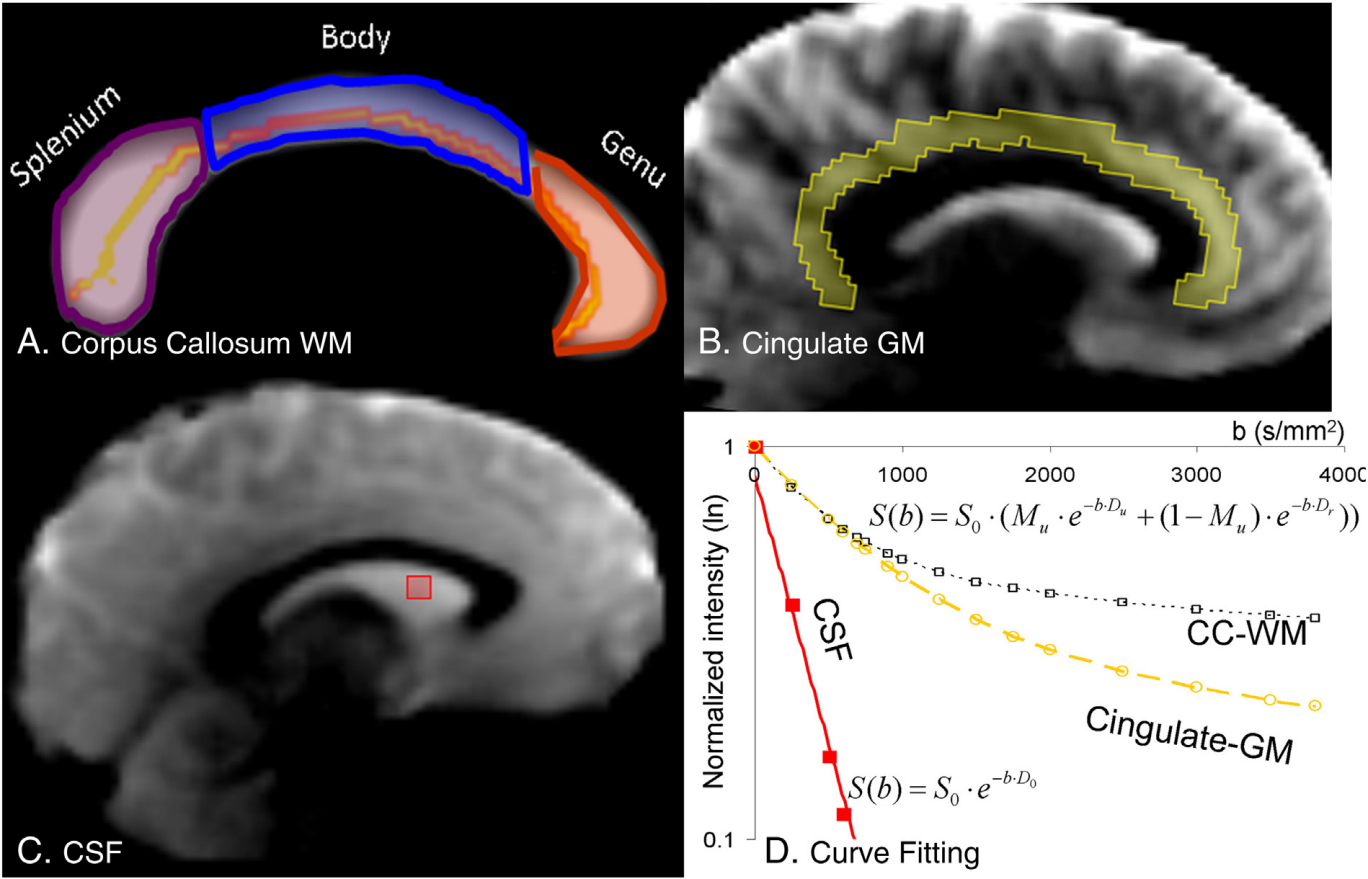
(A) Corpus callosum (CC) white matter (WM) region-of-interest was identified by thresholding FA image at FA = 0.20. The labels for three sub-regions were transferred from population-based, 3D, DTI cerebral WM tract atlas developed in John Hopkins University and distributed with the FSL package (46). The skeleton of cerebral WM was shown on the background of the image. (B) The region of interest for cingulate gray matter (GM) was identified using radial diffusivity maps that show excellent contrast between GM, WM and CSF. (C) A small (5 × 5 × 5 mm) region of interest was placed in the lateral ventricle. (D) The signal decay for WM and GM was best described by bi-exponential function (r ≅ .98). The signal decay in CSF was mono-exponential.

The MBI protocol was developed based on q-space protocols for in-vivo mapping of water diffusion in the brain (Clark et al., 2002; Wu et al., 2011b). This protocol consisted of 15 shells of b-values (b = 250, 500, 600, 700, 800, 900, 1000, 1250, 1500, 1750, 2000, 2500, 3000, 3500 and 3800 s/mm^2^; diffusion gradient duration = 47 ms, diffusion gradient separation = 54 ms). Thirty isotropically distributed diffusion weighted directions were collected per shell, including sixteen b = 0 images. Three dummy scans preceded the data collection to establish the steady state. The highest b-value (b = 3800 s/mm^2^) was chosen because the SNR for the corpus callosum in the average diffusion image (SNR = 6.1 ± 0.7) measured in five healthy volunteers (ages 25–50 years) during protocol development, approached the empirically selected lower limit of SNR = 5.0. The b-values and the number of directions per shell were chosen for improved fit of the bi-exponential model and signal to noise ratio (Jones et al., 1999). The imaging data were collected using a single-shot, echo-planar, single refocusing spin-echo, T2-weighted sequence with a spatial resolution of 1.7 × 1.7 × 4.6 mm and seven slices prescribed in sagittal orientation to sample the midsagittal band of the corpus callosum (Fig. 1). The sequence control parameters were TE/TR = 120/1500 ms with the FOV = 200 mm. The total scan time was about 10 min per subject.

#### 3.1.1 Bi-exponential modeling of diffusion decay

The MBI images were pre-processed to perform a region-of-interest (ROI) based fit a two-compartment diffusion model (Eq. (1)) that assumed that intravoxel signal is formed by a contribution from two compartments (Clark et al., 2002; Panagiotaki et al., 2009; Sukstanskii et al., 2003; Wu et al., 2011b).

(1) $$S\left(b\right)=S_{0}\cdot \left(M_{u}\cdot e^{-b\cdot D_{u}}+\left(1-M_{u}\right)\cdot e^{-b\cdot D_{r}}\right)$$

where S(b) is the average diffusion weighted signal for a given b value, averaged across all directions. M_u_ is the fraction of the signal that comes from the compartment with unrestricted diffusion. The (1 − M_u_) is the fraction of the signal that comes from the compartment with restricted diffusion. D_u_ and D_r_ are the apparent diffusion coefficients of the unrestricted and restricted compartments, respectively. The term ‘compartment’ refers to bi-compartmental modeling analyses, Eq. (1), and does not represent physical compartment or space. The diffusion weighted image for each of the b-values S(b) were calculated for the four ROIs in cerebral white matter: the whole and the genu, body and splenium of corpus callosum and one in cerebral gray matter, the cingulate gyrus (Fig. 1). Segmentation of corpus callosum was performed based on the contrast in the FA values between corpus callosum and the nearby GM and CSF. To perform this segmentation, voxel-wise FA, radial and axial diffusivity images for each subject were created using Camino software (http://cmic.cs.ucl.ac.uk/camino) (Alexander et al., 2011). Individual FA and diffusivity images were resampled to an isotropic resolution of 1 mm. FA images were thresholded at FA = .20 and spatially registered to the corpus callosum segmented from the population-based, 3D, DTI cerebral WM tract atlas developed in John Hopkins University (JHU) (Wakana et al., 2004) using 9-parameter linear spatial normalization (FSL-FLIRT). An experienced neuroanatomist, blind to the diagnostic status, transferred the labels for genu, body and splenium from the atlas to FA images using Mango software (http://ric.uthscsa.edu/Mango). Next, radial diffusivity map was used to delineate the ROI for the cingulate cortex (Fig. 1B). Radial diffusivity images were used because they provided excellent contrast between GM (moderate radial diffusivity), WM (low radial diffusivity) and CSF (high radial diffusivity) (Fig. 1B). Finally, a small (5 × 5 × 5 mm) ROI was placed in the lateral ventricle to measure diffusion decay in CSF. There were no significant patient control differences in the volumes of any ROI (p > .3). The bi-exponential diffusion model (Eq. (1)) was fitted for the WM of corpus callosum and its three subdivision and for the GM of cingulate cortex using non-linear, least square fitting implemented in the [R] package (R-Development-Core-Team, 2009) (Fig. 1D). In CSF, signal decay due to diffusion weighting was mono-exponential (Fig. 1D).

#### 3.1.2 Permeability–diffusivity (PD) model

We used the theoretical model developed by Sukstanskii and colleagues to describe the effect of permeable barriers on diffusion (Sukstanskii et al., 2003, 2004) and to calculate the ratio of D_r_ and D_u_, which we now termed the permeability–diffusivity index (PDI) (Eq. (2))

(2) PDI=DrDu.

**Fig. 2:**
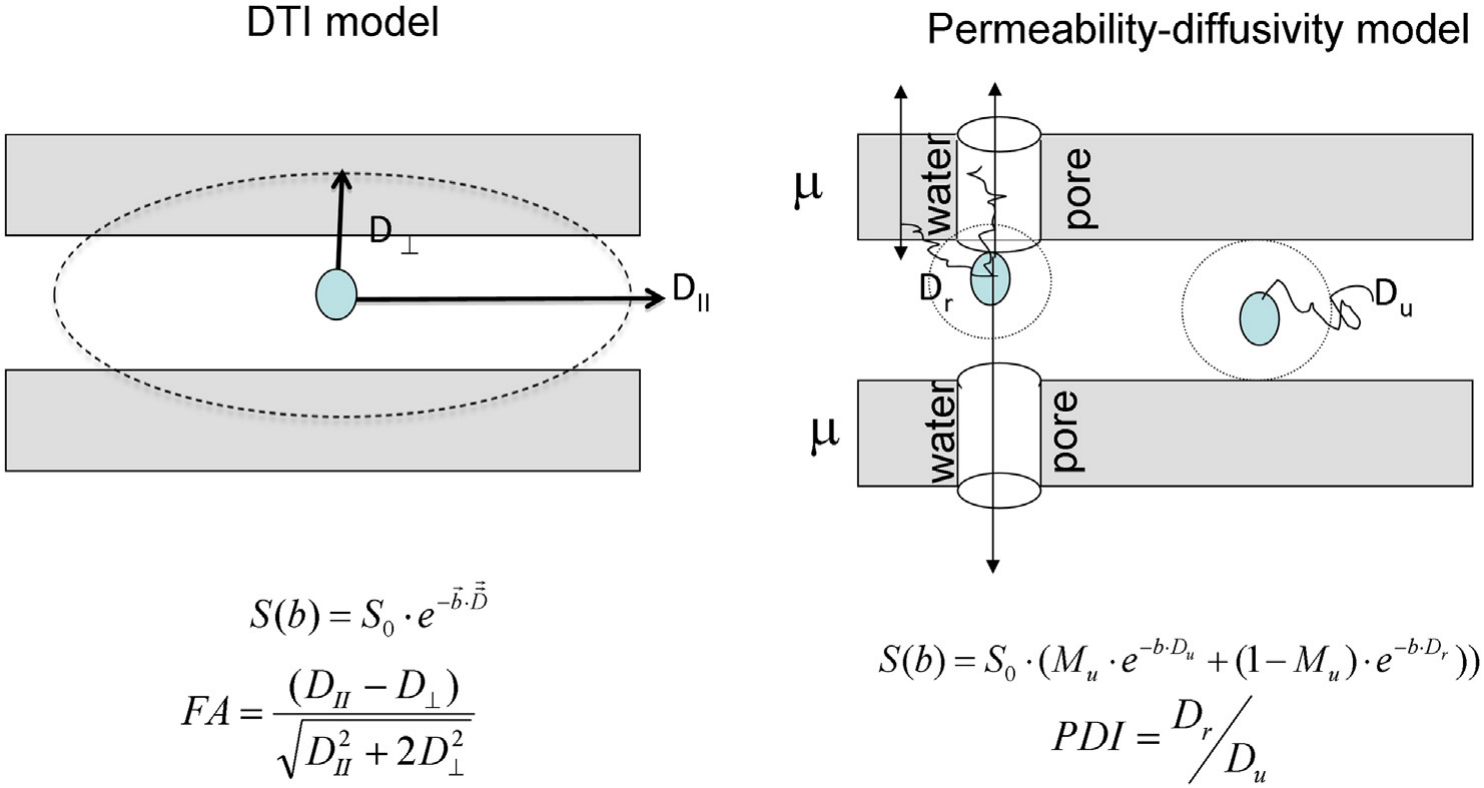
Schematic comparison of the standard DTI model (left) and permeability–diffusivity (PD) model, right. The standard DTI model assumes that signal is produced by single pool of anisotropically diffusing water and characterizes anisotropy of water diffusion using fractional anisotropy (FA). PD-model, developed by Sukstanskii (34), assumes that the signal is produced by two quasi-pools of isotropic diffusing water. Unrestricted pool is produced by water molecules that are sufficiently away from the cellular membranes to be unaffected by them. The water near the membrane forms the restricted pool whose diffusivity depends on both passive diffusivity (thin arrow) of water through cellular/myelin membrane and active (thick arrow) permeability via are the ionic channels and water pores that use water as substrate for compartment exchange.

Fig. 2 provides a schematic diagram to clarify the PD-model parameters in the context of cerebral WM and compare them to the standard DTI model. Standard DTI model assumes that the diffusion signal is formed by a single pool of water with anisotropic diffusion properties. Diagonalization of diffusion tensor produces eigenvalues that correspond to diffusivities along axial (D_II_) and radial (D_⊥_) directions. Fractional anisotropy (FA) is a weighted combination of D_II_ and D_⊥_ to characterize the directional sensitivity of water diffusion.

The PD-model posits that two quasi-pools of water are caused by an inhomogeneous distribution of local transverse in the presence of permeable membrane. The unrestricted pool is characterized by the diffusion coefficient D_u_ and the ‘population fraction’ M_u_. It is formed by water molecules that are sufficiently far away from axonal walls or other water barriers to be unaffected by them. The restricted pool, characterized by D_r_ and ‘population fraction’ (1 − M_u_), is formed by water that is sufficiently close to cellular walls to encounter diffusivity barriers. According to PD-model, small changes of the axonal or cellular membrane permeability, within the normal physiological range, mainly impact the restricted diffusion coefficient (D_r_) (Sukstanskii et al., 2003, 2004) and this makes PDI to be sensitive to membrane permeability (μ) (Sukstanskii et al., 2003, 2004). Under normal physiological condition, the axonal and cellular membranes are semi-permeable by both passive exchange (diffusion) and/or active exchange via ionic and water pumps (white pores in Fig. 2) that use water as the substrate for cross-compartment exchange (Baslow, 2002). The impact of active permeability on diffusivity is demonstrated by animal ischemic stroke models. Failure of molecular pumps, following ischemia, leads to a rapid (within minutes) drop in apparent diffusivity (20–50%) and its restoration following reperfusion (Li et al., 2000). This is in contrast to changes in structural indexes, such as FA, which may take hours to manifest (Li et al., 2000).

We hypothesized that PDI should provide a complementary description of the diffusivity in the white matter compared with traditional DTI measures (radial diffusivity, axial diffusivity, or FA) all of which use a simple ellipsoidal model of diffusivity within a uniformly restricted space. In comparison, PDI should incorporate information of the relative strength of the diffusivity within restricted and unrestricted spaces, which should theoretically derive a more accurate description of the white matter integrity than FA.

### 3.2 HARDI protocol

The details of imaging protocol are described elsewhere (Kochunov et al., 2011b). In short, diffusion tensor data were collected using a single-shot, echo-planar, single refocusing spin-echo, T2-weighted sequence with a spatial resolution of 1.7 × 1.7 × 3.0 mm. The sequence parameters were: TE/TR = 87/8000 ms, FOV = 200 mm, axial slice orientation with 50 slices and no gaps, 64 isotropically distributed diffusion weighted directions, two diffusion weighting values (b = 0 and 700 s/mm^2^) and five b = 0 images. These parameters were calculated using an optimization technique that maximizes the contrast to noise ratio for FA measurements (Jones et al., 1999). The total scan time was about 9 min per subject.

The HARDI data was processed using a tract-based spatial statistics (TBSS) method, distributed as a part of FMRIB Software Library (FSL) package (Smith et al., 2006) as described elsewhere (Kochunov et al., 2011b). The population-based, 3D, DTI cerebral WM tract atlas developed in John Hopkins University and distributed with the FSL package (Wakana et al., 2004) was used to calculate population average FA values along the spatial course of major WM tracts as described elsewhere (Kochunov et al., 2011a, 2012).

## 4 Statistical analysis

Patient-control differences on the PDI and FA were evaluated using a two-tail t-test. Group-related differences in aging trends were evaluated using linear regression to replicate previously reported finding of accelerated aging decline in FA in patients (Kochunov et al., 2013). These analyses were performed for the whole-corpus callosum and for the genu because that region of corpus callosum was shown to have the largest patient–control and aging-related differences (Kochunov et al., 2013).

Next, linear correlation analysis was used to establish the relationship between PDI and the three standard DTI parameters (D_II_, D_⊥_, FA) derived from the HARDI data. Again, this analysis was performed on the entire corpus callosum and the genu of corpus callosum. The significance threshold for group differences was adjusted for multiple comparisons using Bonferroni correction. Previous research demonstrated bi-exponential diffusion decay in cortical GM (Clark et al., 2002). We explored the inter-group differences in PD-parameters calculated from the cingulate cortex that overlays corpus callosum.

## 5 Results

Patient and control groups showed no significance difference in age, gender, BMI or current smoking status (Table 1). The bi-exponential model provided an excellent fit for the diffusion decay, as the function of b-values, in both WM and GM in patients and controls (average r^2^ ≅ .98 vs. .65 for bi vs. mono-exponential approximation, respectively), while diffusion decay in CSF was mono-exponential (r^2^ ≅ .97) (Fig. 1D).

### 5.1 Comparing DTI and MBI measures on group effects

Patient group had significantly lower apparent WM diffusivity coefficient of the restricted compartment (D_r_) (p = 0.0036) and PDI coefficients (p = 0.0007) for the corpus callosum (Table 2). Patients also had significantly lower average corpus callosum FA values (p = 0.03). There were no significant group-wise differences in other diffusion measurements (Table 2). Regional analysis demonstrated that the genu of corpus callosum showed the most significant group-wise differences on PDI parameters (Table 3). Genu was also the only partition of corpus callosum that showed significant difference in FA values (Table 3). The effect sizes on group difference of PDI at the genu (Cohen's d = 1.14) was about twice that of the FA at the genu (d = 0.52), suggesting that PDI and FA both identified white matter abnormalities in schizophrenia while PDI more than doubled the effect size in differentiating the two groups.

### 5.2 Evaluation of age-related trends

**Fig. 3:**
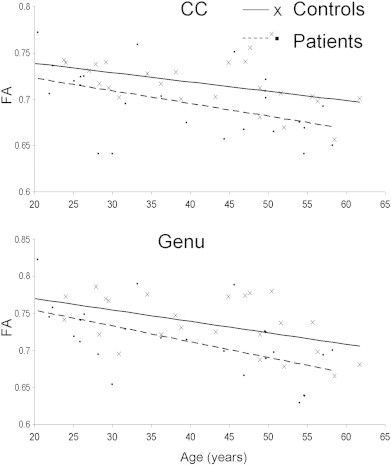
Age-related trends for the FA values for the whole CC (top) and genu (bottom). FA showed an age related decline in both groups in the CC (controls, FA = 1.1 E − 3 ∗ Age + 0.759; r = 0.40; p = 0.03 vs. patients PDI = 1.6 E − 4 ∗ Age + 0.75; r = 0.43; p = 0.02) and the genu (controls, FA = 1.5 E − 3 ∗ Age + 0.80; r = 0.47; p = 0.01 vs. patients PDI = 2.5 E − 4 ∗ Age + 0.79; r = 0.56; p = 0.003).

**Fig. 4:**
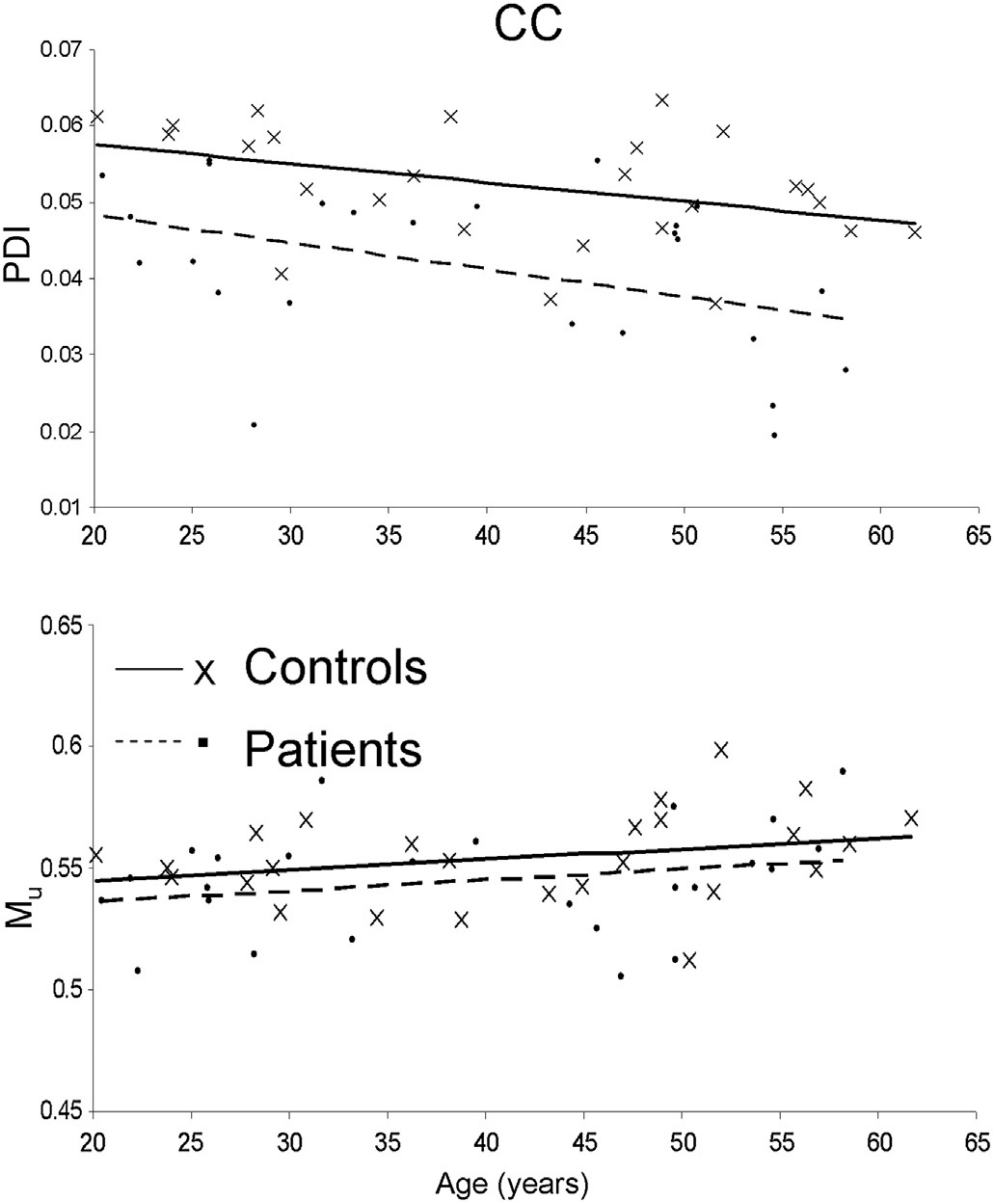
Age-related trends for the PDI and M_u_ for the whole corpus callosum. PDI showed an age related decline in patients but not controls (patients PDI = 3.3 E − 4 ∗ Age + 0.0535; r = 0.41; p = 0.04; controls, PDI = 2.0 E − 4 ∗ Age + 0.0525; r = 0.33; p = 0.08). M_u_ showed no significant age-related increase in the CC (patients M_u_ = 5.3 E − 4 ∗ Age + 0.52; r = 0.24; p = 0.25; controls, M_u_ = 5.1 E − 4 ∗ Age + 0.53; r = 0.26; p = 0.24).

FA is reliably correlated with age during development and senescence (Kochunov et al., 2012) and our data showed the expected age-related decline in FA values for both corpus callosum and the genu (Fig. 3). PDI showed similar trends: PDI for the whole corpus callosum showed a significant age related decline in patients (β = 3.3 × 10^− 4^; r = .41, p = .04) and this trend approached significance in controls (β = 2.0 × 10^− 4^;r = .33, p = .08) (Fig. 4). The age-related decline in PDI of the genu was significant in both groups (β = 2.5 × 10^− 4^; r = .37, p = .05 and β = 4.3 × 10^− 4^; r = .43, p = .03 for controls and patients, respectively) (Fig. S1, see Supplement).

### 5.3 Correlation of PDI and FA

**Fig. 5:**
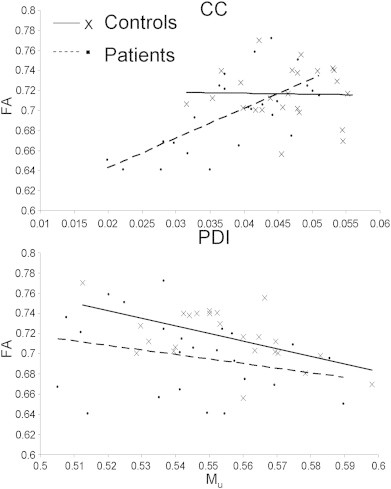
Plots of FA values versus PDI (top) and M_u_ (bottom) for the whole CC. CC FA was highly correlated with PDI patients but not controls (patients FA = 2.96 ∗ PDI + 0.58; r = 0.67; p = 1 E − 4; controls, FA = − 0.09 ∗ PDI + 0.72; r = 0.02; p = 0.9). FA showed a significant negative association with M_u_ in controls but not patients (controls, FA = − 0.75 ∗ M_u_ + 1.13; r = 0.55; p = 0.002 vs. patients FA = − 0.45 ∗ M_u_ + 0.94; r = 0.26; p = 0.20).

PDI was significantly correlated with FA in patients but not controls in either whole-corpus callosum (r = .67 vs. − .02) or genu (r = .72 vs. .03) and this difference in trends was significant (p = .01 and .002, for the whole corpus callosum and genu, respectively) (Fig. 5 and S2, Supplement).

### 5.4 Group differences in the PDI in the gray matter

FA characterizes anisotropy of water diffusion and is not commonly used in GM where the diffusion is isotropic, with some exceptions (Kroenke et al., 2009). Diffusion signal in GM shows the same bi-exponential dependence on b-values (Clark et al., 2002) and therefore PDI may theoretically be sensitive to the restrictive effects of neuronal membrane on diffusivity. We explored if PDI calculated from the cingulate cortex that overlays corpus callosum (Fig. 1) could be informative to group-wise differences. Similar to WM, patients showed significantly reduced PDI than controls (.067 ± .014 vs. .081 ± .011; p = .001; for patients and controls respectively). The difference in M_u_ was also not significant (M_u_ = .66 ± .02 and .67 ± .02, p = .5 for patients and controls, respectively).

## 6 Discussion

We examined the effect of schizophrenia on permeability–diffusivity (PD) parameters derived from bi-exponential modeling of cerebral diffusion signals. We observed that schizophrenia patients had a significantly (p ~ 10^− 3^) reduced permeability–diffusivity-index (PDI) in both WM of corpus callosum and GM of cingulate cortex when compared to normal controls. The most significant differences (p = 1 × 10^− 4^) were observed for the genu of corpus callosum. Patients also had significantly reduced FA values for the whole brain and the corpus callosum (p = .03 for both). Therefore, while both PDI and FA measurements identified patient-control differences the effect size of PDI measurements was significantly more robust. Combined with theoretical formulation of PD-model by Sukstanskii and colleagues (Sukstanskii et al., 2004), these data suggest that schizophrenia may be associated with restricted permeability of axonal and neural cell membranes in both WM and GM compartments.

We used parameters derived from the theoretical PD-model by Sukstanskii and colleagues that suggested that the bi-exponential decay of diffusion signal is sensitive to permeability of cellular membranes. Earlier studies had ascribed the bi-exponential diffusion decay to intra-axonal or extra-cellular compartments (Assaf and Cohen, 1998; Assaf et al., 2002, 2005; Hwang et al., 2003), however, recent studies and computer simulations argued against it (Hwang et al., 2003; Schwarcz et al., 2004; Stokes et al., 2012; Yablonskiy et al., 2003). Instead, the two exponential components do not have a clear physical meaning but rather approximate a more complex functional signal behavior (Sukstanskii et al., 2004). The modeling efforts by Sukstanskii demonstrated that, in the range of membrane permeability observed in cerebral WM (μ_eff_ = 0.01–0.1; μ_eff_ = μ × d / D_0_, where μ is the flux through axonal membrane = 0.1–1 mm/s, d is the average distance of membrane bound compartment ~ 10^− 3^ mm and D_0_ is diffusivity of free water ~ 10^− 2^ mm^2^/s), both PDI and M_u_, are sensitive to membrane's permeability. The PDI changes rapidly (1000%) within the range of the normal physiological permeability (Fig. S3, see Supplement) (Sukstanskii et al., 2003, 2004) while the range of change in M_u_ is modest (~ 10%) (Fig. S3, see Supplement). One outcome of this modeling is the demonstration of strong dependence of PDI on permeability of cellular membranes. Since the PD-model approximates cerebral WM as a simple, periodic spatial structure, it likely is too basic to explain all the trends. Therefore we attempted to clarify biological meaning of our findings by exploring association between PD-parameter, age and other imaging traits.

Of the imaging parameters examined, FA showed the most robust age related decline. Moreover, FA in patients declined at twice the rate in controls. This is consistent with findings of accelerated WM aging in schizophrenia by this group (Kochunov et al., 2013). PDI showed a significant age-related decline in patients, while the PDI decline in controls was only significant in the genu. The slope of age-related decline in the PDI in patients was also about twice that in controls. This suggested that both FA and PDI are sensitive to aging effects of WM and perhaps to those aspects that contribute to the accelerated aging effect in the patients.

**Fig. 6:**
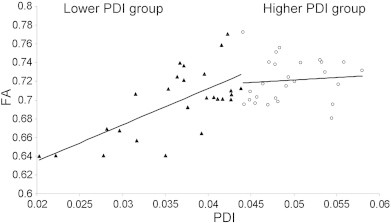
Plots of FA values versus PDI for CC for all subjects based on the median split, of PDI values. FA and PDI values were highly correlated in the lower PDI group (FA = 3.86 ∗ PDI + 0.54; r = 0.7; p = 1 E − 5) but not in not in the other half (FA = 0.55 ∗ PDI + 0.69; r = 0.02; p = 0.9). The lower PDI group also had significantly lower FA values (p = 0.008) but showed no difference in age (p = 0.2).

Correlation between MBI and DTI parameters provided additional insight into the physiological significance of PD-parameters. The PDI and FA measurements were derived from different sequences and analysis models and therefore provide independent assessment of WM integrity. Notably, PDI was significantly correlated with FA values in patients but not in controls. The biological cause for this separation between patients and controls correlation remains uncertain. Closer inspection revealed that this trend was only present in subjects with low PDI values. In fact, performing an even split of all subjects, regardless of the diagnosis, using the PDI of the corpus callosum demonstrated a highly significant positive correlation between PDI and FA (r^2^ = .48, p < 10^− 5^) in the bottom half. This group consisted of nineteen patients and seven controls. The correlation between PDI and FA was not significant (r^2^ = 0.11; p = 0.10) in the upper half (Fig. 6). There was no significant difference in age between two groups (average age = 43.4 ± 12.2 vs 37.3 ± 12.3 for lower and upper PDI groups, respectively; p = 0.09). However, the control subjects in lower PDI group were significantly older than controls in upper PDI group (average age = 49.7 ± 8.1 vs 38.2 ± 12.5 for lower and upper PDI groups, respectively; p = 0.04). This suggests that the correlation between PDI and FA values is not specific to the schizophrenia patients but was also present in older normal individuals.

**Fig. 7:**
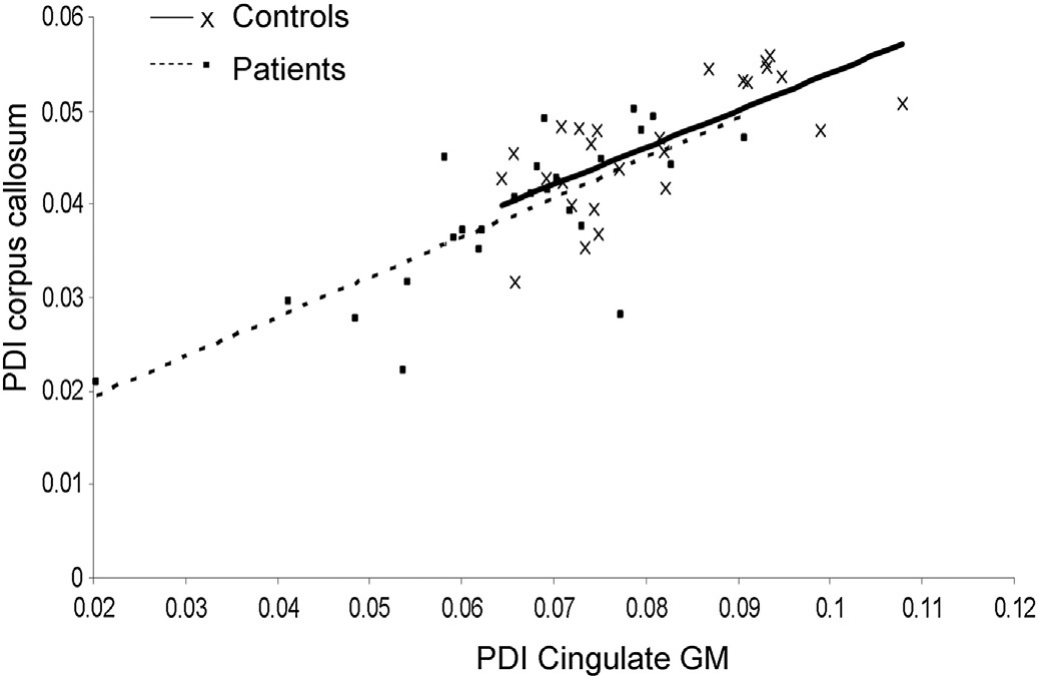
Plots of PDI for cingulate GM vs corpus callosum WM showed a highly significant trend in both groups (controls, PDI CC = 0.40 ∗ PDI GM + 0.01; r = 0.7; p = 1 E − 5; patients, PDI CC = 0.42 ∗ PDI GM + 0.01; r = 0.73; p = 1 E − 5).

Finally, we observed that the PDI for cingulate GM shared equally high (~ 50%) variability with the PDI in corpus callosum in both groups (Fig. 7). In contrast, the GM's M_u_ was not significantly correlated (p > .4) with the WM's M_u_ in either group. Cingulate PDI showed age-related decline, similar to that observed in corpus callosum (r^2^ = .28 and .08; p = 0.006 and 0.15 for controls and patients, respectively). The age-related trend in the cingulate M_u_ (r^2^ = .05 and .11; p = 0.3 and 0.10) was the opposite (decline vs. rise) to the age-related increase observed in M_u_ measured in WM. Adding to the tissue-specific difference, cingulate PDI was not correlated with the FA values in the corpus callosum in either group (r^2^ = .05 and .09; p > .1 for controls and patients, respectively). This suggests that GM and WM share significant variability in PDI but the trends in M_u_ appear to be tissue specific.

The design of this study does not allow determination on how the results may be related to the etiology of schizophrenia. We also cannot reject the long-term effects of chronic antipsychotic medication. However, the correlation between chlorpromazine dose equivalents of antipsychotic medication was robustly non-significant for all PD or FA traits (p > .30). Additionally, PDI and M_u_ coefficients in three un-medicated patients were no different from the medicated patients (all p > 0.9). Nonetheless, the finding of reduced PDI in patients was robust and statistically significant and had a strong association with aging and classical DTI parameters. Long imaging time limited PD-analyses a small number of slices. We chose the sagittal band of corpus callosum because of consistent patient–control differences in its white matter integrity, reported for this region. Presently, the PD-model does not account for crossing fibers and it remains to be determined how crossing fibers may influence this model. Therefore, using corpus callosum in our first clinical application overcame this limitation since corpus callosum has a simple architecture with no crossing fibers (Aboitiz et al., 1992). Multiplex DTI sequences, including these distributed by the Human Connectom Project (Feinberg et al., 2010; Moeller et al., 2010), can accelerate collection of imaging data by 2 to 8 fold, making the whole-brain MBI practical (< 20 min). Additional development of PD model that accounts for crossing fibers will therefore be important.

## 7 Conclusions

We aimed to evaluate the application of MBI and PD-analysis technique to schizophrenia, and compared them with traditional DTI measurements. Theoretical formulations from the Sukstanskii et al.'s model were used to explain the significantly reduced PDI in patients as evidence for restricted permeability of cellular membranes. Much research is needed to understand the biological underpinnings of the findings here. Nevertheless, the PDI measurement, especially in WM, demonstrated a much bigger effect size in schizophrenia than DTI-FA, and theoretical modeling of this approach indicated that patients with this disorder may have reduced active molecular ion and water pump driven permeability.

## Figures and Tables

**Table 1:** Participants' demographic and clinical information including age, age-of-onset, duration of the disorder, body mass index (BMI), the current smoking status, and scores on the Brief Psychiatric Rating Scale (BPRS) for schizophrenia patients. Group-wise significance was calculated using a two-tailed t-test.

	Average age, range (years)	Age-of-onset (years)	Duration (years)	BMI	Current smokers	BPRS
Patients (7 F/19 M)	39.9 ± 12.8, 20–59	18.2 ± 7.4	19.3 ± 13.4	28.9 ± 5.0	66%	1.9 ± 0.4
Controls (12 F/15 M)	37.5 ± 11.9, 20–62	N/A	N/A	27.2 ± 5.5	46%	N/A
Group difference, p-value	0.8	N/A	N/A	0.2	0.1	N/A

**Table 2:** Patient–control differences on the permeability–diffusivity (PD) and diffusion tensor imaging (DTI) trait measurement of corpus callosum. Group-wise significance was calculated using a two-tailed t-test.

	PD-measurements: corpus callosum				DTI measurements: corpus callosum		
	M_u_	D_u_ (mm^2^/s)	D_r_ (mm^2^/s)	PDI	FA	D_II_ (mm^2^/s)	D_⊥_ (mm^2^/s)
Patients	.55 ± .03	1.8 ± .2 × 10^− 3^	7.1 ± 1.5 × 10^− 5^	3.8 ± 1.1 × 10^− 2^	.69 ± .03	1.3 ± .0.1 × 10^− 3^	5.0 ± 0.5 × 10^− 4^
NC	.55 ± .02	1.8 ± 0.1 × 10^− 3^	8.4 ± 1.5 × 10^− 5^	4.6 ± .08 × 10^− 2^	.72 ± .03	1.4 ± .1 × 10^− 3^	4.8 ± 0.5 × 10^− 4^
p-Value	0.8	0.4	0.0036	7.0 × 10^− 4^	.03	0.3	0.1

**Table 3:** Patient–control differences in the PD and DTI-FA measurements for three regions of corpus callosum. Significance was calculated using a two-tailed t-test. Bolded values are significant after correction for multiple (N = 12) comparisons.

	Genu				Body				Splenium				DTI-FA		
	M_u_	D_u_ (mm^2^/s)	D_r_ (mm^2^/s)	PDI	M_u_	D_u_ (mm^2^/s)	D_r_ (mm^2^/s)	PDI	M_u_	D_u_ (mm^2^/s)	D_r_ (mm^2^/s)	PDI	Genu	Body	Splenium
Patients	.58 ± .03	1.6 ± .1 × 10^− 3^	6.7 ± 1.6 × 10^− 5^	4.1 ± 1.1 × 10^− 2^	.55 ± .03	1.8 ± .3 · 10^− 3^	8.0 ± 2.5 × 10^− 5^	4.4 ± 1.1 × 10^− 2^	.51 ± .02	2.0 ± .2 × 10^− 3^	6.5 ± .1 × 10^− 5^	3.2 ± .7 × 10^− 2^	.71 ± .05	.63 ± .06	.74 ± .03
NC	.58 ± .02	1.7 ± 0.1 × 10^− 3^	8.3 ± 1.7 × 10^− 5^	5.2 ± .08 × 10^− 2^	.56 ± .02	1.7 ± .2 × 10^− 3^	9.2 ± 1.7 × 10^− 5^	5.3 ± .08 × 10^− 2^	.51 ± .02	2.1 ± 0.2 × 10^− 3^	7.8 ± 0.2 × 10^− 5^	3.7 ± .6 × 10^− 2^	.73 ± .03	.66 ± .04	.76 ± .02
p-Value	0.6	0.02	.0019	.0001	0.4	0.2	0.05	.0033	0.9	0.2	0.002	.00091	.04	.07	.10

## Appendix A Supplementary data

Supplementary figures. Figure S1. Age-related trends for the PDI (top) and Mu (bottom) for the genu of corpus callosum. PDI showed an age related decline in both groups (controls, PDI = 2.5E−4 * Age + 0.0541; r = 0.37; p = 0.05 vs. patients PDI = 4.3E−4 * Age + 0.0497; r = 0.43; p = 0.03). M_u_ showed no significant age-related increase (controls, Mu = 1.1E−3 * Age + 0.541; r = 0.47; p = 0.01 vs. patients Mu = 1.5E−3 * Age + 0.539; r = 0.44; p = 0.02). Figure S2. Plots of FA values versus PDI (top) and Mu (bottom) for the genu of corpus callosum. Genu FA was strongly correlated to PDI in patients and but not in controls (patients FA = 2.51 * PDI + 0.59; r = 0.72; p = 1E−5; controls, PDI = 0.61 * FA + 0.68; r = 0.10; p = 0.5). CC FA showed a significant negative association with Mu in controls but not patients (controls, FA = −0.57 * M0 + 1.0; r = 0.57; p = 0.001 vs. patients FA = −0.24 * M0 + 0.83; r = 0.25; p = 0.22). Figure S3. Modeling of PDI and Mu based on the theoretical work by Sukstanskii (34) for range of membrane permeability observed in cerebral WM (meff = 0.01– 0.1 where meff = m · d/D0 and m is the flux through axonal membrane under normal physiological conditions (0.1–1 mm/sec), d is the average distance of membrane bound compartment ~10^– 3^ mm and D0 is the diffusivity of free water ~10^– 2^ mm^2^/s). This modeling demonstrates that both PDI and Mu, are sensitive to membrane's permeability, however, PDI changes are more dynamic over the normal permeability range than these in Mu.
