# Supplementary material for: Permeability–diffusivity modeling vs. fractional anisotropy on white matter integrity assessment and application in schizophrenia
Source: Neuroimage Clin. 2013 Jul 11;3:18–26. doi: 10.1016/j.nicl.2013.06.019 (PMC3791292; doi:10.1016/j.nicl.2013.06.019)
Supplement: Supplementary file 1 — Supplementary figures. Figure S1. Age-related trends for the PDI (top) and Mu (bottom) for the genu of corpus callosum. PDI showed an age related decline in both groups (controls, PDI = 2.5E−4 * Age + 0.0541; r = 0.37; p = 0.05 vs. patients PDI = 4.3E−4 * Age + 0.0497; r = 0.43; p = 0.03). Mu showed no significant age-related increase (controls, Mu = 1.1E−3 * Age + 0.541; r = 0.47; p = 0.01 vs. patients Mu = 1.5E−3 * Age + 0.539; r = 0.44; p = 0.02). Figure S2. Plots of FA values versus PDI (top) and Mu (bottom) for the genu of corpus callosum. Genu FA was strongly correlated to PDI in patients and but not in controls (patients FA = 2.51 * PDI + 0.59; r = 0.72; p = 1E−5; controls, PDI = 0.61 * FA + 0.68; r = 0.10; p = 0.5). CC FA showed a significant negative association with Mu in controls but not patients (controls, FA = −0.57 * M0 + 1.0; r = 0.57; p = 0.001 vs. patients FA = −0.24 * M0 + 0.83; r = 0.25; p = 0.22). Figure S3. Modeling of PDI and Mu based on the theoretical work by Sukstanskii (34) for range of membrane permeability observed in cerebral WM (meff = 0.01– 0.1 where meff = m · d/D0 and m is the flux through axonal membrane under normal physiological conditions (0.1–1 mm/sec), d is the average distance of membrane bound compartment ~10– 3 mm and D0 is the diffusivity of free water ~10– 2 mm2/s). This modeling demonstrates that both PDI and Mu, are sensitive to membrane's permeability, however, PDI changes are more dynamic over the normal permeability range than these in Mu. [file mmc1.doc]

**Permeability-diffusivity modeling vs. fraction anisotropy on white matter integrity assessment and application in schizophrenia**

**Kochunov P.1,2, Chiappelli J. 1, Hong L.E.1,.**

Maryland Psychiatric Research Center, Department of Psychiatry, University of Maryland School of Medicine, Baltimore.

Department of Physics, University of Maryland Baltimore County.

*Corresponding Author: (pkochunov@mprc.umaryland.edu)

Address:

Maryland Psychiatric Research Center

Department of Psychiatry

University of Maryland School of Medicine, Baltimore, MD, USA

Phone: (410) 402-6110

Fax (410) 502-6778


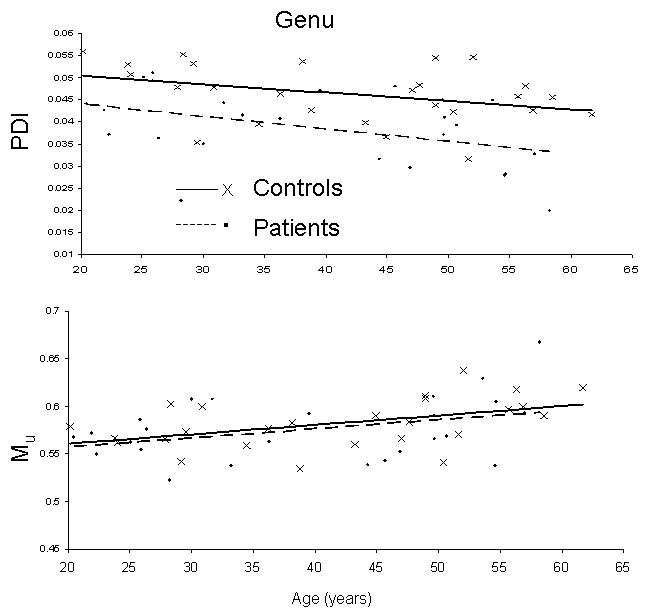


Figure S1. Age-related trends for the PDI (top) and Mu (bottom) for the genu of corpus callosum. PDI showed an age related decline in both groups (controls, PDI=2.5 E-4*Age + 0.0541; r=0.37; p=0.05 vs. patients PDI=4.3E-4*Age + 0.0497; r=0.43; p=0.03). M_u_ showed no significant age-related increase (controls, Mu=1.1E-3*Age + 0.541; r=0.47; p=0.01 vs. patients Mu=1.5E-3*Age + 0.539; r=0.44; p=0.02).


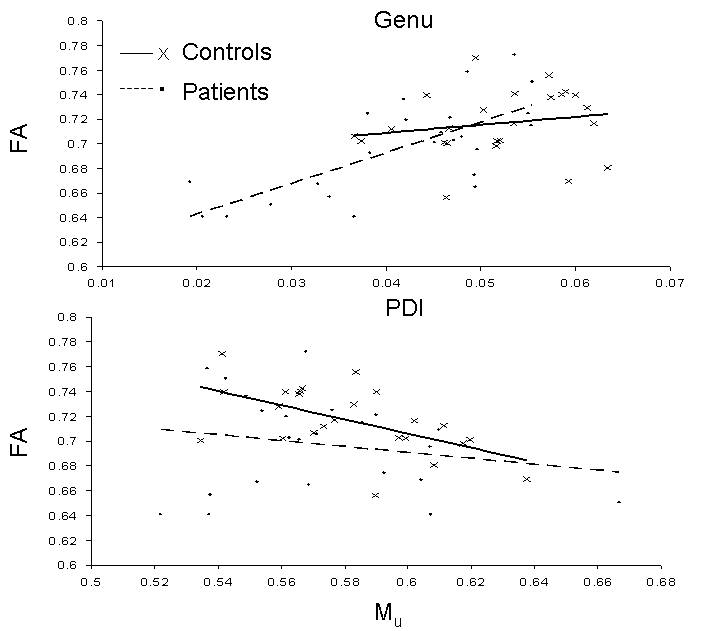


Figure S2. Plots of FA values versus PDI (top) and Mu (bottom) for the genu of corpus callosum. Genu FA was strongly correlated to PDI in patients and but not in controls (patients FA=2.51*PDI + 0.59; r=0.72; p=1E-5; controls, PDI=0.61*FA + 0.68; r=0.10; p=0.5). CC FA showed a significant negative association with Mu in controls but not patients (controls, FA=-0.57*M0 + 1.0; r=0.57; p=0.001 vs. patients FA=-0.24*M0 + 0.83; r=0.25; p=0.22)


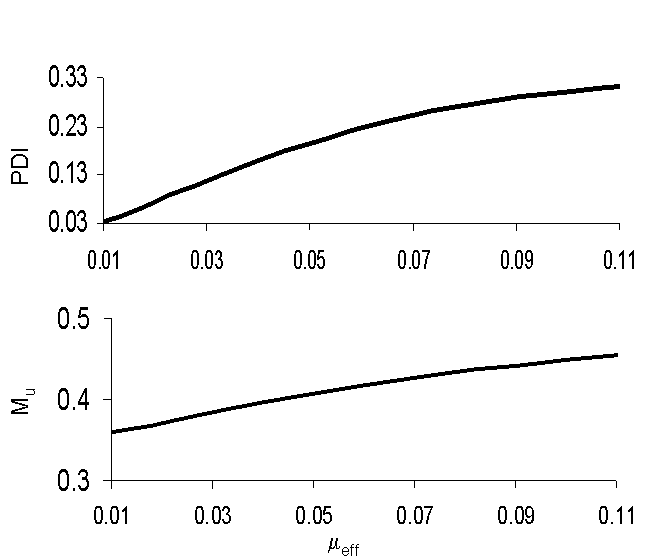


Figure S3. Modeling of PDI and Mu based on the theoretical work by Sukstanskii (34) for range of membrane permeability observed in cerebral WM (μeff=0.01-0.1 where μeff=μ·d/D0 and μ is the flux through axonal membrane under normal physiological conditions (0.1-1mm/sec), d is the average distance of membrane bound compartment ~10^-3^ mm and D0 is diffusivity of free water ~10^-2^ mm2/s). This modeling demonstrate that both PDI and Mu, are sensitive to membrane’s permeability, however, PDI changes are more dynamic over the normal permeability range then these in Mu
